# Internal consistency of the self-reporting questionnaire-20 in occupational groups

**DOI:** 10.1590/S1518-8787.2016050006100

**Published:** 2016-03-10

**Authors:** Kionna Oliveira Bernardes Santos, Fernando Martins Carvalho, Tânia Maria de Araújo

**Affiliations:** I Departamento de Biofunção. Universidade Federal da Bahia. Salvador, BA, Brasil; II Departamento de Medicina Preventiva. Universidade Federal da Bahia. Salvador, BA, Brasil; III Departamento de Saúde. Universidade Estadual de Feira de Santana. Feira de Santana, BA, Brasil

**Keywords:** Mental Health, Questionnaires, utilization, Reproducibility of Results, Validation Studies, Mental Disorders, diagnosis, Workers

## Abstract

**OBJECTIVE:**

To assess the internal consistency of the measurements of the Self-Reporting Questionnaire (SRQ-20) in different occupational groups.

**METHODS:**

A validation study was conducted with data from four surveys with groups of workers, using similar methods. A total of 9,959 workers were studied. In all surveys, the common mental disorders were assessed via SRQ-20. The internal consistency considered the items belonging to dimensions extracted by tetrachoric factor analysis for each study. Item homogeneity assessment compared estimates of Cronbach’s alpha (KD-20), the alpha applied to a tetrachoric correlation matrix and stratified Cronbach’s alpha.

**RESULTS:**

The SRQ-20 dimensions showed adequate values, considering the reference parameters. The internal consistency of the instrument items, assessed by stratified Cronbach’s alpha, was high (> 0.80) in the four studies.

**CONCLUSIONS:**

The SRQ-20 showed good internal consistency in the professional categories evaluated. However, there is still a need for studies using alternative methods and additional information able to refine the accuracy of latent variable measurement instruments, as in the case of common mental disorders.

## INTRODUCTION

In the early 1970s, the World Health Organization^a^ built the Self-Reporting Questionnaire (SRQ) to assess the impacts of mental health problems in primary health care in periphery countries. This instrument was composed of 30 questions evaluating psycho-emotional symptoms, alcohol abuse, psychotic disorders and seizures. The target population was primary health service users. In 1980^12^, a version with 20 questions (SRQ-20) was developed, covering only psycho-emotional aspects. It was proposed for screening of common mental disorders (CMD), which are nonpsychotic symptoms characterized by insomnia, fatigue, irritability, forgetfulness, trouble concentrating, and somatic complaints^8^.

The SRQ-20 has been widely used and the performance of its measurements has been evaluated in populations of health service users^22^. However, few studies have assessed the validity and consistency of SQR-20 measurements regarding occupation^21^.

Instruments for assessment of behavioral traits or sets of symptoms need item homogeneity to capture different aspects of the same variable. Internal consistency evaluations of instruments are considered a reliability criterion for a measurement. However, this interpretation has its limits because measurements of internal consistency are based on management of the scale at a specific point, not considering sources of variation throughout time or among observers^24^.

The concept of validity is related to the quality of a given measurement. Reliability reflects the set of random and systematic errors inherent in a measurement. The relationship between validity and reliability can be analyzed by the consistency of external (validity) and internal (reliability) criteria^25^.

Reliability reflects a necessary but insufficient condition for the validity of a particular measurement. This condition does not characterize a property of a research instrument; essentially, reliability refers to the ability of the measurement produced by the instrument to be consistent in time and space or among different observers^16^. In addition, the conditions to evaluate reliability are relative, since every measurement has a degree of reliability when applied to a particular population under specific conditions^24^.

In the reliability assessment, three procedures can be adopted, depending on the type of the instrument and form of measurement. The stability evaluation of the measurement considers the consistency over time (test-retest), a technique related to the concept of reproducibility of measurements. The second procedure concerns measurement equivalence by considering different forms of measurement (interobserver). The measurement can also be evaluated by the internal consistency of the set of items used to estimate a certain latent variable or dimension^15^. Thus, the internal consistency of the instrument shows the degree of homogeneity of the measurement when the items or subscales measure the same variable^16^.

This study aimed to assess the internal consistency of SRQ-20 measurements in occupational groups.

## METHODS

A validation study was conducted with four surveys that used similar methodologies in different occupational categories in the state of Bahia, Northeastern Brazil.

Study 1 – Informal workers. An epidemiological survey, with systematic sampling of 1,458 stallholders, street vendors and motorcycle taxi drivers in Feira de Santana, Bahia, in 2008^1^.

Study 2 – Teachers. A census of the 4,496 teachers from the 365 public preschools and elementary schools in Salvador, Bahia, 2006^4^.

Study 3 – Health care workers. A cross section of a multicenter study using the same sampling procedures of a survey carried out in Belo Horizonte^2^, Minas Gerais, among primary health care workers from four municipalities of Bahia (Feira de Santana, Jequié, Santo Antônio de Jesus and a health district of Salvador). Its proportional stratified sampling considered: the distribution of the number of workers by geographical area; definition of events of interest estimates; composition of the sample according to the percentage of workers in each geographical area of the participating municipalities; and the draw, by random procedure, of the workers included in the study in each region. Throughout the 2012-2013 period, 2,448 workers were evaluated.

Study 4 – Urban workers. A random sample of 1,557 individuals, representing workers over 15 years of age, stratified by subdistricts of the urban area of Feira de Santana, in 2007^20^.

In all studies, common mental disorders were evaluated with the use of the Self-Reporting Questionnaire (SRQ-20). To analyze the internal consistency of the dimensions (subscales) of the instrument, a tetrachotric correlation factor analysis was previously performed using the method of principal components with factor extraction, according to the Kaiser criterion of eigenvalue greater than 1. The scree plot technique was used to confirm the amount of factors to be extracted. The items presenting load greater than 0.40 were retained for composition of the factors^11^. PROMAX oblique rotation was applied for better interpretation of the values, using the Stata program, version 11.0.

To compare estimates of internal consistency of subscales (dimensions) extracted by tetrachoric factor analysis, we used alpha (α) values by the Kuder-Richardson formula (KD-20), α for the tetrachoric correlations matrix, and stratified α^6^, to investigate a possible underestimation of internal consistency.

Stratified α was estimated using Microsoft Excel^®^ (2007), employing the formula in which *S* ^2^_i_ is the variance of the items that constitute the factor *i (i = 1, ..., f)*, α_i_ is Cronbach’s alpha for the factor *i* and *S* ^2^_T_ is the total variance of the instrument, according to the equation below. The variation of 0.65-0.90 was adopted as the reference parameter of satisfactory performance^17^.


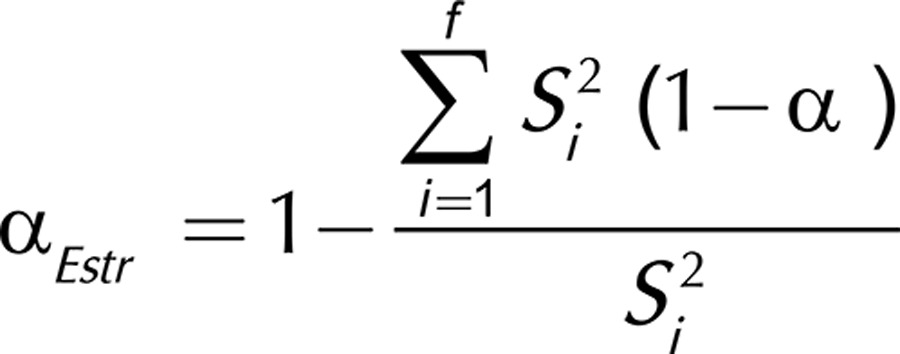


The four studies were evaluated and approved by research ethics committees when they were conducted. The present study was approved by the Research Ethics Committee of the Instituto de Saúde Coletiva da Universidade Federal da Bahia (CAAE 18723813.9.0000.5030).

## RESULTS

Most evaluated workers were female, especially among teachers (92.0%; study 2) and health workers (80.6%; study 3). In the four studies, the predominant age group was 30 to 45 years (Table 1). The educational attainment of the workers differed among studies. Most informal workers (study 1) reported less than primary educational attainment (95.9%). In study 3, 42.9% reported elementary educational attainment and 41.3% secondary technical education or higher education. Secondary technical education or higher education was reported by 82.1% of teachers (study 2) and 55.9% of workers in general (study 4).

**Table 1:** Sociodemographic characteristics of the populations of the four studies. Bahia, Northeastern Brazil, 2006-2013.

Study - Population (N)		n	%
Study 1 – Informal workers (N = 1,458)			
Sex	Female	728	49.9
	Male	730	50.1
Age group (years)	< 30	537	36.8
	30-45	553	37.9
	> 45	368	25.2
Educational attainment (n = 1,438)	Less than primary education	1,379	95.9
	Secondary technical education/Higher education	9	0.6
	No qualification	50	3.5
Study 2 – Teachers (N = 4,496)			
Sex (n = 4,342)	Female	3,994	92.0
	Male	348	8.0
Age group (years) (n = 4,302)	< 30	773	18.0
	30-45	2,289	53.2
	> 45	1,240	28.8
Educational attainment (n = 4,398)	Less than primary education	717	16.3
	Secondary technical education/Higher education	3,609	82.1
	Graduate education	72	1.6
Study 3 – Health care workers (N = 2,448)			
Sex (n = 2,421)	Female	1,951	80.6
	Male	470	19.4
Age group (years) (n = 2,395)	< 30	581	24.3
	30-45	1,071	44.7
	> 45	743	31.0
Educational attainment (n = 2,419)	Less than primary education	1,038	42.9
	Secondary technical education/Higher education	1,000	41.3
	Graduate education	381	15.8
Study 4 – Urban workers (N = 1,557)			
Sex	Female	851	54.7
	Male	706	45.3
Age group (years)	< 30	576	37.0
	30-45	584	37.5
	> 45	397	25.5
Educational attainment (n = 1,269)	Less than primary education	536	42.3
	Secondary technical education/Higher education	710	55.9
	No qualification	23	1.8

Filling of SRQ-20 items varied among studies (Table 2). Study 2 had the greatest number of losses, with 605 missing data (13.5%), followed by study 3, which showed a loss of 60 data (2.4%). Smaller loss percentages were found in studies 1 and 4 (0.3% and 1.2%, respectively).

**Table 2:** Cronbach’s alpha (KD-20) and tetrachoric correlation matrix alpha values of the items composing SRQ-20, according to group of workers. Bahia, Northeastern Brazil, 2006-2013.

	Group of workers							

	Informal workers		Teachers		Health care workers		Urban workers	
			
	(n = 1,453)^a^		(n = 3,891)^b^		(n = 2,397)^c^		(n = 1,539)^d^	

SRQ-20 items	α1^e^	α2^f^	α1^e^	α2^f^	α1^e^	α2^f^	α1^e^	α2^f^
1. Do you often have headaches?	0.85	0.93	0.85	0.93	0.82	0.92	0.84	0.92
2. Is your appetite poor?	0.84	0.93	0.85	0.93	0.82	0.92	0.83	0.92
3. Do you sleep badly?	0.84	0.93	0.85	0.93	0.81	0.91	0.83	0.92
4. Are you easily frightened?	0.84	0.93	0.85	0.93	0.81	0.91	0.83	0.92
5. Do your hands shake?	0.84	0.93	0.85	0.93	0.81	0.91	0.84	0.92
6. Do you feel nervous, tense or worried?	0.84	0.93	0.84	0.93	0.80	0.91	0.83	0.92
7. Is your digestion poor?	0.84	0.92	0.85	0.93	0.81	0.91	0.84	0.92
8. Do you have trouble thinking clearly?	0.84	0.93	0.85	0.93	0.81	0.91	0.83	0.92
9. Do you feel unhappy?	0.84	0.92	0.84	0.93	0.80	0.90	0.83	0.92
10. Do you cry more than usual?	0.84	0.93	0.85	0.93	0.81	0.91	0.83	0.92
11. Do you find it difficult to enjoy your daily activities?	0.84	0.93	0.85	0.93	0.81	0.91	0.83	0.92
12. Do you find it difficult to make decisions?	0.84	0.93	0.85	0.93	0.81	0.91	0.84	0.92
13. Is your daily work suffering?	0.84	0.93	0.85	0.93	0.82	0.91	0.84	0.92
14. Are you unable to play a useful part in life?	0.85	0.93	0.86	0.94	0.82	0.92	0.83	0.92
15. Have you lost interest in things?	0.84	0.92	0.85	0.93	0.81	0.91	0.83	0.92
16. Do you feel that you are a worthless person?	0.85	0.93	0.86	0.93	0.82	0.92	0.84	0.92
17. Has the thought of ending your life been on your mind?	0.85	0.93	0.86	0.94	0.82	0.91	0.84	0.92
18. Do you feel tired all the time?	0.84	0.93	0.84	0.93	0.81	0.91	0.83	0.92
19. Do you have uncomfortable feelings in your stomach?	0.84	0.93	0.85	0.93	0.81	0.91	0.83	0.92
20. Are you easily tired?	0.84	0.93	0.85	0.93	0.81	0.91	0.83	0.92

Total	0.85	0.94	0.85	0.94	0.82	0.92	0.84	0.93

^a^ 0.3% losses.

^b^ 13.5% losses.

^c^ 2.4% losses.

^d^ 1.2% losses.

^e^ Cronbach’s alpha (KD-20), if the item is removed.

^f^ Cronbach’s alpha for the tetrachoric correlation matrix, if the item is removed.

The α values of SRQ-20 dimensions of the occupational groups evaluated showed significant variations. The use of the alpha adjusted by the tetrachoric correlation matrix allowed more robust coefficients, considering the sample size.

In studies 1 and 2, we observed similar KD-20 and tetrachoric matrix α values (0.85 and 0.94, respectively). Study 3 (health workers) showed the lowest value of internal consistency for both estimators (0.82 and 0.92). In study 4, standardized values of the item set were 0.84 for the KD-20 estimate and 0.93 for the tetrachoric correlation α. In all studies, removing items did not change substantially the global values of internal consistency estimates of the instrument.

The factor analysis allowed the extraction of three factors that differed as to classification of the dimensions and number of items in each dimension among studies (Table 3).

**Table 3:** Number of items, number of losses, estimates of Cronbach’s alpha and of stratified Cronbach’s alpha of the SQR-20 dimensions extracted by tetrachoric factor analysis among groups of workers. Bahia, Northeastern Brazil, 2006-2013.

Study - Population (N)	Items (N)	Losses	Cronbach’s alpha	Cronbach’s alpha (Tetrachoric)
Study 1 – Informal workers (N = 1,453)				
F1 - Depressive mood/Anxiety symptoms	11	5	0.79	0.90
F2 - Somatic component	5	0	0.65	0.79
F3 - Decreased energy	4	0	0.65	0.76
Scale total	20	5	0.85	0.92
Stratified Cronbach’s alpha	-	-	0.86	0.93
Study 2 – Teachers (N = 3,891)				
F1 - Decreased energy/Anxiety symptoms	11	434	0.82	0.92
F2 - Somatic component	6	295	0.68	0.82
F3 - Depressive mood	3	242	0.41	0.74
Scale total	20	605	0.85	0.93
Stratified Cronbach’s alpha	-	-	0.82	0.94
Study 3 – Health care workers (N = 2,397)				
F1 - Depressive mood	9	33	0.67	0.87
F2 - Decreased energy/Anxiety symptoms	9	44	0.72	0.87
F3 - Somatic component	2	17	0.70	0.87
Scale total	20	60	0.82	0.92
Stratified Cronbach’s alpha	-	-	0.83	0.93
Study 4 – Urban workers (N = 1,539)				
F1 - Somatic component/Anxiety symptoms	11	8	0.78	0.88
F2 - Decreased energy	5	6	0.62	0.82
F3 - Depressive mood	4	4	0.64	0.86
Scale total	20	18	0.84	0.93
Stratified Cronbach’s alpha	-	-	0.85	0.93

The Cronbach’s α estimated for tetrachoric correlation matrix produced higher values for all dimensions of the studies. The internal consistency of the instrument items, assessed by stratified Cronbach’s α, was high (> 0.80) in most studies.

In study 1, the dimension represented by factor 1 (F1 - depressive mood or anxiety symptoms) concentrated the largest number of items (11) and higher internal consistency according to both estimation methods (0.79 and 0.90). Factor 2 (F2) represented the somatic dimension and factor 3 (F3), the dimension “decreased energy”. These last two dimensions showed identical Cronbach’s α values (0.65), but differed in estimates assessed for tetrachoric matrix correlation, with a higher value for F2 (α = 0.79).

Among teachers (study 2), dimension “depressive mood” presented limitations in internal consistency (α = 0.41). However, this same dimension stayed within the reference standard (α = 0.74) when the tetrachoric matrix was considered.

The three dimensions extracted by factor analysis in study 3 presented similar estimates of reliability (α = 0.87) when the tetrachoric matrix was considered.

Study 4 showed the smallest reliability estimates among SRQ-20 dimensions. Factor 2 (“decreased energy”) and factor 3 (“depressive mood”) presented Cronbach’s α coefficients below the reference value (α = 0.62 and α = 0.64, respectively). However, when using the method of tetrachoric correlation matrix, all factors (dimensions) reached coefficients greater than 0.80.

## DISCUSSION

Internal consistency estimates of SRQ-20 dimensions and global scores, using a tetrachoric correlation matrix, showed adequate values consistent with the literature^17^. However, the high proportion of losses in study 2 may have compromised Cronbach’s alpha estimates. Both losses affecting α values and differences among occupational groups show that the reliability of a measurement, evaluated by the reproducibility or homogeneity of measurement items, cannot be interpreted as an inherent or immutable property of an instrument because it depends on the interaction between the instrument and a specific assessed group^24^.

Internal consistency has been considered a suitable measurement to describe traits, characteristics of behaviors or disorders in a particular context, but not necessarily to identify groups possessing such attributes or not^25^. Thus, group characteristics may affect the homogeneity of measurement items. Intra- or inter-subject differences related to the variability of the data affects the α value. Generally, the smaller the variability of intra-subject responses and the larger the variability of inter-subject responses, the greater the α value^16^.

In this study, most occupational groups had a predominance of females, with an average age of 30 to 45 years and differences in educational attainment. Low educational attainment can be a barrier to express emotional disorders^7^. However, the stratification used here hampers a detailed analysis of this characteristic in the interpretation of the scale.

In addition to the differences between the groups, the high degree of consistency in the measurements of a scale denotes ease of interpretation of the final score, as a reflection of the items composing the instrument. To do this, the items should be moderately correlated with each other and maintain a correlation with the total score of scale^13^.

The interpretation of internal consistency measurements produced by SRQ-20 has been discussed in the literature. Most studies use Cronbach’s α (KD-20) as a reference measurement to evaluate the consistency of SRQ-20^9^ ^,^ ^14^ ^,^ ^21^ ^,^ ^22^. The total scores of the instrument have been interpreted as satisfactory; however, when evaluating the dimensions that represent groups of symptoms, Cronbach’s α coefficients show smaller values. The multidimensionality of the variable measured by SRQ-20 limits homogeneous relations among items and justifies the irregular performance of internal consistency instrument measurements^21^.

Some aspects should be considered when evaluating the results related to internal consistency estimates of an instrument. Uncritical acceptance of α values, as a reflex of high levels of internal consistency, can lead to impaired judgment of the real scale homogeneity.

The α value depends not only on the magnitude of the correlation of the items in a scale, but also on the number of items that compose it. Thus, the greater the number of items in a scale, the greater the estimated internal consistency. Another condition that requires greater caution when interpretating internal consistency is the combination of scales that assess independent constructs, because the increase in the items will elevate α estimates. In addition, high α values may be related to redundancy of the scale items, compromising content validity, since an item set can assess the same conditions in different ways^25^.

In this study, most dimensions extracted by tetrachoric factor analysis showed internal consistency estimates within the assumed reference parameter (α = 0.65-0.90). However, the parameters used as reference for the estimates of α are also the target of criticism.

There are several reference values to evaluate the internal consistency of scales^17^ ^,^ ^21^. The variations in reference values are due to different numbers of scale items and size of the investigated samples. Generally, scales with fewer than 10 items, small samples, and α = 0.70 are considered of good internal consistency in their measurements. On the other hand, if the scale has more items and a sample size greater than 300, α = 0.90 must be used as a parameter^25^. Despite the proposed parameters, critical evaluations must be stimulated. Estimates of α are affected by the amount of items and dimensionality of the scales. Values higher than 0.90 suggest redundancy of scale items and a need to reduce the instrument^26^.

This study provides evidence to characterize SRQ-20 as a multidimensional instrument, with dimensions varying among the different occupational groups investigated. Estimates of α cannot be interpreted as a property of the instrument, since they are conditioned by scale scores in a given population^24^.

In general, one-dimensional measurements feature high levels of internal consistency (high homogeneity). However, high internal consistency values represent conditions necessary but insufficient to ensure the unicity of a scale^5^. Thus, multidimensional instruments featuring high internal consistency levels for a particular measurement show that, although there are different dimensions, the components are strongly interrelated^16^.

The α values may underestimate the true internal consistency of the measurementss of a multidimensional instrument because the α estimate suggests equivalent distinction among the questionnaire items^18^ ^,^ ^19^ ^,^ ^b^. Congeneric scales, characterized by correlation of items among themselves, are also affected by the underestimation of alpha values^10^. For multidimensional instruments, the stratified α has showed better performance for the estimates than conventional estimators, although the differences are not so expressive^b^. Therefore, the principle of tau equivalence must be considered when analyzing estimates of Cronbach’s α among multidimensional instruments. In these cases, the assumption that each item of test measures the same latent trait in an instrument is violated by the dimensions extracted by factor analysis. In this way, the α values of a multidimensional instrument will be underestimated^26^.

In the context of psychometric assessments, we highlight the debate on the superiority of measurements used as reliability criteria^3^. Cronbach’s α measurements are widely used uncritically and often considered a reference for scale reliability. However, it is necessary to deepen the analysis of α values and to compare the indexes based on repetition of measurements (test-retest)^10^.

Operational and theoretical limitations reflect a low threshold of internal consistency for estimates produced by Cronbach’s α. There are also the difficulties of interpretation and judgement of its measurements. Alpha estimates correlate with other statistics, which can confuse results when very low and very high values of this coefficient are found in one-dimensional or multidimensional instruments. Thus, additional information is required to assess α estimates separatedly as an internal consistency measurement^23^.

Despite the extensive use of Cronbach’s α in the internal consistency evaluation of SRQ-20, the discussion about the real implications of its measurements for multidimensional instruments is still recent. Studies are needed to critically assess Cronbach’s α estimates, comparing them with alternative methods and additional information to refine the accuracy of latent variable measurement instruments, as in the case of mental disorders.

## Footnotes

a World Health Organization, Division of Mental Health. User’s guide to self-reporting questionnaire (SRQ). Geneva: World Health Organization; 1994.

b Widhiarso W. Estimate reliability measurement for multidimensional scales. Social Science Research Network; 2009 [cited 2015 Jul 28]. Available from: http://papers.ssrn.com/sol3/papers.cfm?abstract_id=1597532
