# Supplementary material for: Internal consistency of the self-reporting questionnaire-20 in occupational groups
Source: Rev Saude Publica. 2016 Mar 10;50:6. doi: 10.1590/S1518-8787.2016050006100 (PMC4794769; doi:10.1590/S1518-8787.2016050006100)
Supplement: Translation

# Translation: Consistência interna do *self-reporting questionnaire*-20 em grupos ocupacionais

**Authors:** Kionna Oliveira Bernardes Santos, Fernando Martins Carvalho, Tânia Maria de Araújo

**Affiliations:** I Departamento de Biofunção. Universidade Federal da Bahia. Salvador, BA, Brasil; II Departamento de Medicina Preventiva. Universidade Federal da Bahia. Salvador, BA, Brasil; III Departamento de Saúde. Universidade Estadual de Feira de Santana. Feira de Santana, BA, Brasil

**Keywords:** Saúde Mental, Questionários, utilização, Reprodutibilidade dos Testes, Estudos de Validação, Transtornos Mentais, diagnóstico, Trabalhadores

## Abstract

**OBJETIVO:**

Avaliar a consistência interna das medidas do *Self-Reporting Questionnaire* (SRQ-20) em diferentes grupos ocupacionais.

**MÉTODOS:**

Realizou-se estudo de validação com dados de quatro inquéritos de grupos de trabalhadores, utilizando procedimentos metodológicos semelhantes. Foram estudados 9.959 trabalhadores. Em todos os inquéritos, os transtornos mentais comuns foram avaliados por meio do SRQ-20. A consistência interna considerou os itens pertencentes às dimensões extraídas por análise fatorial tetracórica, para cada estudo. A avaliação da homogeneidade dos itens comparou estimativas alpha de Cronbach (KD-20), o alpha aplicado a uma matriz de correlações tetracóricas e do alpha de Cronbach estratificado.

**RESULTADOS:**

As dimensões do SRQ-20 apresentaram valores adequados, considerando os parâmetros de referência. A consistência interna dos itens do instrumento, avaliada pelo escore do alpha de Cronbach estratificado, foi elevada (> 0,80) nos quatro estudos.

**CONCLUSÕES:**

O SRQ-20 apresentou boa consistência interna nas categorias profissionais avaliadas. Entretanto, ainda são necessários estudos que utilizem métodos alternativos e informações adicionais que possam refinar a precisão dos instrumentos de mensuração de construtos latentes, como no caso dos transtornos mentais comuns.

## INTRODUÇÃO

Na década de 1970, Organização Mundial de Saúde^a^ construiu o *Self-Reporting Questionnaire* (SRQ) para avaliar os impactos dos problemas de saúde mental na atenção básica à saúde em países periféricos. O instrumento foi composto por 30 questões que avaliavam sintomas psicoemocionais, uso abusivo de álcool, transtornos psicóticos e crise convulsiva, tendo como população-alvo usuários dos serviços primários de saúde. Em 1980^12^, foi desenvolvida uma versão com 20 questões (SRQ-20), abordando apenas aspectos psicoemocionais, proposta para triagem dos transtornos mentais comuns (TMC). Os TMC são sintomas não psicóticos, caracterizados por insônia, fadiga, irritabilidade, esquecimento, dificuldade de concentração e queixas somáticas^8^.

O SRQ-20 tem sido amplamente utilizado e o desempenho de suas medidas tem sido avaliado em populações de usuários de serviços de saúde^22^. Entretanto, poucos estudos avaliaram a validade e a consistência das medidas do SRQ-20 em âmbito ocupacional^21^.

Em instrumentos de avaliação de traços de comportamentos ou conjunto de sintomas, é necessário haver homogeneidade nos seus itens para que diferentes aspectos de um mesmo construto sejam captados. Avaliações de consistência interna dos itens de instrumentos são consideradas critério de confiabilidade de uma medida. Entretanto, esta interpretação tem limites, uma vez que as medidas de consistência interna são baseadas em administração pontual da escala, não considerando fontes de variação ocorridas no tempo ou entre observadores^24^.

O conceito de validade está relacionado à qualidade de determinada mensuração. A confiabilidade reflete o conjunto de erros aleatórios e sistemáticos inerentes a uma medida. A relação existente entre validade e confiabilidade pode ser analisada por consistência presente em critérios externos (validade) e internos (fidedignidade)^25^.

A confiabilidade reflete uma condição necessária, mas não suficiente, para a validade de determinada mensuração. Esta condição não caracteriza uma propriedade de um instrumento de pesquisa; essencialmente, a confiabilidade refere-se à capacidade de a medida produzida pelo instrumento ser consistente no tempo e no espaço ou entre diferentes observadores^16^. Além disso, as condições para avaliar confiabilidade são relativas, visto que toda mensuração possui um grau de confiabilidade, quando aplicada a determinada população em condições específicas^24^.

Na avaliação da confiabilidade, três procedimentos podem ser adotados, a depender do tipo instrumento e da forma de mensuração. A avaliação da estabilidade da medida considera a consistência ao longo do tempo (teste-reteste), técnica relacionada ao conceito de reprodutibilidade das medidas. O segundo procedimento é relativo à equivalência da medida, ao considerar diferentes formas de mensuração (interobservadores). A medida pode, ainda, ser avaliada pela consistência interna do conjunto de itens utilizados para estimar determinado construto ou dimensão latente^15^. Assim, a consistência interna do instrumento sinaliza o grau de homogeneidade da medida, quando os itens ou subescalas componentes mensuram o mesmo construto^16^.

Este estudo objetivou avaliar a consistência interna das medidas do SRQ-20 em grupos ocupacionais.

## MÉTODOS

Foi realizado estudo de validação envolvendo quatro inquéritos que utilizaram metodologias semelhantes em diferentes categorias profissionais no estado da Bahia.

Estudo 1 – Trabalhadores informais. Inquérito epidemiológico, com amostragem sistemática de 1.458 feirantes, ambulantes e mototaxistas de Feira de Santana, BA, 2008^1^.

Estudo 2 – Professores. Censo dos 4.496 professores das 365 escolas de ensino infantil e fundamental da rede pública de Salvador, BA, 2006^4^.

Estudo 3 – Trabalhadores da Saúde. Recorte de estudo multicêntrico, utilizando os mesmos procedimentos amostrais de um inquérito realizado em Belo Horizonte^2^, MG, entre trabalhadores da atenção básica de quatro municípios da Bahia (Feira de Santana, Jequié, Santo Antônio de Jesus e um distrito de saúde de Salvador). Foi realizada amostra estratificada proporcional que considerou: a distribuição do número de trabalhadores por área geográfica; definição das estimativas dos eventos de interesse; composição percentual da amostra segundo o número de trabalhadores em cada área geográfica dos municípios participantes e o sorteio, por procedimento aleatório, dos trabalhadores incluídos no estudo em cada região estabelecida. Foram avaliados 2.448 trabalhadores, no período de 2012-2013.

Estudo 4 – Trabalhadores urbanos. Amostra aleatória de 1.557 indivíduos, representando os trabalhadores com mais de 15 anos de idade, estratificados por subdistritos da zona urbana de Feira de Santana, em 2007^20^.

Em todos os estudos, os transtornos mentais comuns foram avaliados com o uso do *Self-Reporting Questionnaire* (SRQ-20). Para análise da consistência interna das dimensões (subescalas) do instrumento, previamente realizou-se análise fatorial de correlações tetracóricas, adotando-se o método de componentes principais com extração de fatores, obedecendo ao critério de Kaiser do autovalor (*eingenvalue*) maior que 1. A técnica de *scree plot* foi utilizada para a confirmação da quantidade de fatores a serem extraídos. Os itens que apresentaram carga superior a 0,40 foram retidos para a composição dos fatores^11^. A rotação oblíqua PROMAX foi aplicada para melhor interpretação dos valores, usando-se o programa Stata, versão 11.0.

Para comparação das estimativas de consistência interna das subescalas (dimensões) extraídas pela análise fatorial tetracórica, foram utilizados os valores de alpha (α) pela fórmula de Kuder-Richardson (KD-20), o α para matriz de correlações tetracóricas e o α estratificado^6^, com o objetivo de investigar a possível subestimação da consistência interna.

O cálculo do α estratificado foi realizado com auxílio do Microsoft Excel^®^ (2007), usando a fórmula em que *S* ^2^_i_ é a variância dos itens que constituem o factor *i (i = 1, ..., f)*, α_i_ é o alpha de Cronbach para o fator *i* e *S* ^2^_T_ é a variância do total do instrumento, conforme equação abaixo). Como parâmetro de referência de desempenho satisfatório^17^, adotou-se a variação de 0,65 a 0,90.



Os quatro estudos foram avaliados e aprovados por comitês de ética em pesquisa no período em que foram realizados. O presente estudo foi aprovado Comitê de Ética em Pesquisa do Instituto de Saúde Coletiva da Universidade Federal da Bahia (CAAE 18723813.9.0000.5030).

## RESULTADOS

A maioria dos trabalhadores avaliados era do sexo feminino, principalmente entre os professores (92,0%; estudo 2) e trabalhadores da saúde (80,6%; estudo 3). Nos quatro estudos, a faixa etária predominante foi a de 30 a 45 anos (Tabela 1). A escolaridade dos trabalhadores diferiu entre os estudos. A maioria dos trabalhadores informais (estudo 1) referiu nível de escolaridade elementar (95,9%). No estudo 3, 42,9% referiram nível fundamental e 41,3% nível técnico ou superior. O nível técnico ou superior foi referido por 82,1% dos professores (estudo 2) e por 55,9% dos trabalhadores em geral (estudo 4).

**Tabela 1:** Características sociodemográficas das populações dos quatro estudos. Bahia, 2006-2013.

Estudo – População (N)		n	%
Estudo 1 – Trabalhadores informais (N = 1.458)			
Sexo	Feminino	728	49,9
	Masculino	730	50,1
Faixa etária	< 30	537	36,8
	30-45	553	37,9
	> 45	368	25,2
Escolaridade (n = 1.438)	Elementar	1.379	95,9
	Técnico/Superior	9	0,6
	Sem qualificação	50	3,5
Estudo 2 – Professores (N = 4.496)			
Sexo (n = 4.342)	Feminino	3.994	92,0
	Masculino	348	8,0
Faixa etária (n = 4.302)	< 30	773	18,0
	30-45	2.289	53,2
	> 45	1.240	28,8
Escolaridade (n = 4.398)	Elementar	717	16,3
	Técnico/Superior	3.609	82,1
	Pós-graduação	72	1,6
Estudo 3 – Trabalhadores da Saúde (N = 2.448)			
Sexo (n = 2.421)	Feminino	1.951	80,6
	Masculino	470	19,4
Faixa etária (n = 2.395)	< 30	581	24,3
	30-45	1.071	44,7
	> 45	743	31,0
Escolaridade (n = 2.419)	Elementar	1.038	42,9
	Técnico/Superior	1.000	41,3
	Pós-graduação	381	15,8
Estudo 4 – Trabalhadores urbanos (N = 1.557)			
Sexo	Feminino	851	54,7
	Masculino	706	45,3
Faixa etária	< 30	576	37,0
	30-45	584	37,5
	> 45	397	25,5
Escolaridade (n = 1.269)	Elementar	536	42,3
	Técnico/Superior	710	55,9
	Sem qualificação	23	1,8

O preenchimento dos itens do SRQ-20 variou segundo os estudos (Tabela 2). O maior número de perdas foi identificado no estudo 2, com 605 dados faltantes (13,5%), seguido pelo estudo 3, que apresentou perda de 60 dados (2,4%). Menores percentuais de perdas foram encontrados nos estudos 1 e 4 (0,3% e 1,2%), respectivamente.

**Tabela 2:** Valores de alpha de Cronbach (KD-20) e alpha de matriz de correlações tetracóricas dos itens que compõem o SRQ-20, segundo grupo de trabalhadores. Bahia, 2006-2013.

	Grupo de trabalhadores							

	Trabalhadores informais		Professores		Trabalhadores da saúde		Trabalhadores urbanos	
			
	(n = 1.453)^a^		(n = 3.891)^b^		(n = 2.397)^c^		(n = 1.539)^d^	

Itens do SRQ-20	α1^e^	α2^f^	α1^e^	α2 ^f^	α1^e^	α2 ^f^	α1^e^	α2 ^f^
1. Tem dores de cabeça frequentemente?	0,85	0,93	0,85	0,93	0,82	0,92	0,84	0,92
2. Você tem falta de apetite?	0,84	0,93	0,85	0,93	0,82	0,92	0,83	0,92
3. Você dorme mal?	0,84	0,93	0,85	0,93	0,81	0,91	0,83	0,92
4. Assusta-se com facilidade?	0,84	0,93	0,85	0,93	0,81	0,91	0,83	0,92
5. Tem tremores nas mãos?	0,84	0,93	0,85	0,93	0,81	0,91	0,84	0,92
6. Sente-se nervoso, tenso ou preocupado?	0,84	0,93	0,84	0,93	0,80	0,91	0,83	0,92
7. Você tem má digestão?	0,84	0,92	0,85	0,93	0,81	0,91	0,84	0,92
8. Tem dificuldade de pensar claramente?	0,84	0,93	0,85	0,93	0,81	0,91	0,83	0,92
9. Sente-se triste ultimamente?	0,84	0,92	0,84	0,93	0,80	0,90	0,83	0,92
10. Tem chorado mais que o costume?	0,84	0,93	0,85	0,93	0,81	0,91	0,83	0,92
11. Tem dificuldade de satisfação com suas tarefas?	0,84	0,93	0,85	0,93	0,81	0,91	0,83	0,92
12. Tem dificuldade de tomar decisão?	0,84	0,93	0,85	0,93	0,81	0,91	0,84	0,92
13. O seu trabalho traz sofrimento?	0,84	0,93	0,85	0,93	0,82	0,91	0,84	0,92
14. Sente-se incapaz de desempenhar papel útil?	0,85	0,93	0,86	0,94	0,82	0,92	0,83	0,92
15. Tem perdido o interesse pelas coisas?	0,84	0,92	0,85	0,93	0,81	0,91	0,83	0,92
16. Sente-se inútil em sua vida?	0,85	0,93	0,86	0,93	0,82	0,92	0,84	0,92
17. Tem pensado em dar fim à sua vida?	0,85	0,93	0,86	0,94	0,82	0,91	0,84	0,92
18. Sente-se cansado todo o tempo?	0,84	0,93	0,84	0,93	0,81	0,91	0,83	0,92
19. Sente desconforto estomacal?	0,84	0,93	0,85	0,93	0,81	0,91	0,83	0,92
20. Você se cansa com facilidade?	0,84	0,93	0,85	0,93	0,81	0,91	0,83	0,92
Total	0,85	0,94	0,85	0,94	0,82	0,92	0,84	0,93

^a^ Perdas de 0,3%.

^b^ Perdas de 13,5%.

^c^ Perdas de 2,4%.

^d^ Perdas de 1,2%.

^e^ Alpha de Cronbach (KD-20), se o item for retirado.

^f^ Alpha de Cronbach para matriz de correlações tetracóricas, se o item for retirado.

Os valores de α das dimensões do SRQ-20 dos grupos ocupacionais avaliados apresentaram variações expressivas. A utilização do alpha ajustado pela matriz de correlações tetracórica permitiu coeficientes mais robustos, considerando o tamanho da amostra.

Nos estudos 1 e 2, observaram-se valores de KD-20 e do α de matriz tetracóricas semelhantes (0,85 e 0,94, respectivamente). O estudo 3 (trabalhadores da saúde) apresentou o menor valor de consistência interna para ambos os estimadores (0,82 e 0,92). No estudo 4, os valores padronizados do conjunto de itens foi de 0,84 para a estimativa KD-20 e 0,93 para o α de correlações tetracóricas. Em todos os estudos, a retirada de itens não alterou substancialmente os valores globais das estimativas de consistência interna do instrumento.

A análise fatorial permitiu a extração de três fatores que diferiram quanto à classificação das dimensões e o número de itens em cada dimensão entre os estudos (Tabela 3).

**Tabela 3:** Número de itens, número de perdas, estimativas do alpha de Cronbach e do alpha de Cronbach estratificado das dimensões do SRQ-20 extraídas pela análise fatorial tetracórica entre grupos de trabalhadores. Bahia, 2006-2013.

Estudo – População (N)	Itens (N)	Perdas	Alpha de Cronbach	Alpha de Cronbach (Tetracórica)
Estudo 1 – Trabalhadores informais (N = 1.453)				
F1 – Humor depressivo/Sintomas de ansiedade	11	5	0,79	0,90
F2 – Componente somático	5	0	0,65	0,79
F3 – Decréscimo de energia	4	0	0,65	0,76
Total da escala	20	5	0,85	0,92
Alpha de Cronbach estratificado	-	-	0,86	0,93
Estudo 2 – Professores (N = 3.891)				
F1 – Decréscimo de energia/Sintomas de ansiedade	11	434	0,82	0,92
F2 – Componente somático	6	295	0,68	0,82
F3 – Humor depressivo	3	242	0,41	0,74
Total da escala	20	605	0,85	0,93
Alpha de Cronbach estratificado	-	-	0,82	0,94
Estudo 3 – Trabalhadores da saúde (N = 2.397)				
F1 – Humor depressivo	9	33	0,67	0,87
F2 – Decréscimo de energia/Sintomas de ansiedade	9	44	0,72	0,87
F3 – Componente somático	2	17	0,70	0,87
Total da escala	20	60	0,82	0,92
Alpha de Cronbach estratificado	-	-	0,83	0,93
Estudo 4 – Trabalhadores urbanos (N = 1.539)				
F1 – Componente somático/Sintomas de ansiedade	11	8	0,78	0,88
F2 – Decréscimo de energia	5	6	0,62	0,82
F3 – Humor depressivo	4	4	0,64	0,86
Total da escala	20	18	0,84	0,93
Alpha de Cronbach estratificado	-	-	0,85	0,93

O coeficiente de α de Cronbach calculado para matriz de correlações tetracóricas produziu valores superiores para todas as dimensões dos estudos participantes. A consistência interna dos itens do instrumento, avaliada pelo α de Cronbach estratificado, foi elevada (> 0,80) na maioria dos estudos.

No estudo 1, a dimensão representada pelo fator 1 (F1 – humor depressivo ou sintomas de ansiedade) concentrou o maior número de itens (11) e apresentou maior consistência interna segundo os dois métodos de estimação (0,79 e 0,90). O fator 2 (F2) representou a dimensão somática e o fator 3 (F3), a dimensão “decréscimo de energia”. Estas duas últimas dimensões apresentaram valores idênticos de α de Cronbach (0,65), mas diferiram nas estimativas avaliadas para correlação de matriz tetracórica, com maior valor para o F2 (α = 0,79).

Entre os professores (estudo 2), a dimensão “humor depressivo” apresentou limitações na consistência interna (α = 0,41). Entretanto, a mesma dimensão manteve-se no padrão de referência ao ser avaliada considerando a matriz tetracórica (α = 0,74).

As três dimensões extraídas pela análise fatorial no estudo 3 apresentaram estimativas de confiabilidade similares (α = 0,87), quando considerada a correlação tetracórica.

O estudo 4 exibiu as menores estimativas de confiabilidade entre as dimensões do SRQ-20. O fator 2 (“decréscimo de energia”) e o fator 3 (“humor depressivo”) apresentaram coeficientes de α de Cronbach abaixo do valor de referência α = 0,62 e α = 0,64, respectivamente. Entretanto, ao utilizar o método de matriz de correlação tetracórica todos os fatores (dimensões) atingiram valores de coeficiente maiores que 0,80.

## DISCUSSÃO

As estimativas de consistência interna das dimensões e os escores globais do SRQ-20, feitas com matriz de correlações tetracóricas, mostraram valores adequados e condizentes com a literatura^17^. Entretanto, a elevada proporção de perdas no estudo 2 pode ter comprometido as estimativas do alpha de Cronbach. Tanto as perdas que afetam os valores de α quanto as diferenças encontradas entre os grupos ocupacionais refletem que a confiabilidade de uma medida, avaliada pela reprodutibilidade ou homogeneidade dos itens de mensuração, não pode ser interpretada como propriedade inerente ou imutável de um instrumento, pois depende da interação entre o instrumento e um grupo específico avaliado^24^.

A consistência interna tem sido considerada medida apropriada para descrever traços, características de comportamentos ou desordens em determinado contexto, mas não necessariamente para discriminar grupos que possuam tais atributos ou não^25^. Assim, características do grupo podem afetar a homogeneidade dos itens de mensuração. Diferenças intrasujeitos ou intersujeitos relacionadas à variabilidade dos dados afetam o valor de α. De uma forma geral, quanto menor a variabilidade das respostas intrasujeitos e maior a variabilidade das respostas intersujeitos, maior o valor de α^16^.

Neste estudo, a maioria dos grupos ocupacionais era do sexo feminino, com faixa etária média (30 a 45 anos) e com diferenças no nível de escolaridade. A baixa escolaridade pode ser uma barreira para expressão de desordens emocionais^7^. Entretanto, a estratificação aqui utilizada não permite análise detalhada desta característica na interpretação da escala.

Para além das diferenças entre os grupos, o alto grau de consistência das medidas de uma escala denota facilidade de interpretação do escore final, como reflexo dos itens que compõem o instrumento. Para isso, os itens devem estar moderadamente correlacionados entre si e manterem correlação com o escore total da escala^13^.

A interpretação das medidas de consistência interna produzidas pelo SRQ-20 tem sido discutida na literatura. A maioria dos estudos usam o α de Cronbach (KD-20) como medida de referência na avaliação da consistência do SRQ-20^9^ ^,^ ^14^ ^,^ ^21^ ^,^ ^22^. Os escores totais do instrumento têm sido interpretados como satisfatórios; entretanto, ao avaliar as dimensões que representam grupos de sintomas, os coeficientes α de Cronbach apresentam menores valores. A multidimensionalidade do construto avaliado pelo SRQ-20 limita relações homogêneas entre os itens e justifica o desempenho irregular da consistência interna das medidas do instrumento^21^.

Alguns aspectos devem ser considerados na avaliação dos resultados relativos às estimativas da consistência interna de um instrumento. A aceitação acrítica dos valores de α, como reflexo de altos níveis de consistência interna, podem implicar problemas de julgamento da real homogeneidade da escala.

O valor de α não depende apenas da magnitude da correlação dos itens de uma escala, mas, também, do número de itens que a compõem. Assim, quanto maior o número de itens em uma escala, maior será a estimativa de consistência interna. Outra condição que necessita maior cautela na interpretação da consistência interna é a junção de escalas que avaliam construtos independentes, pois o aumento dos itens elevará as estimavas de α. Além disso, altos valores de α podem implicar em redundância dos itens da escala, comprometendo a validade de conteúdo, dado que um conjunto de itens pode avaliar as mesmas condições de diferentes formas^25^.

Neste estudo, a maioria das dimensões extraídas pela análise fatorial tetracórica apresentou estimativas de consistência interna dentro do parâmetro de referência assumido (α = 0,65-0,90). Entretanto, os parâmetros utilizados como referência para as estimativas de α também são alvo de crítica.

Existem diversos valores de referência para avaliação da consistência interna de escalas^17^ ^,^ ^21^. As variações nos valores de referência devem-se a diferentes números de itens da escala e ao tamanho das amostras investigadas. De maneira geral, escalas com menos de 10 itens, com tamanho amostral reduzido e que apresentem valores de α = 0,70, são consideradas com boa consistência interna em suas medidas. Em contrapartida, caso a escala possua número maior de itens e tamanho amostral maior que 300, o valor de α = 0,90 deve ser utilizado como parâmetro^25^. Apesar dos parâmetros propostos, avaliações críticas devem ser estimuladas. Estimativas de α são afetadas pela quantidade de itens e dimensionalidade das escalas. Valores superiores a 0,90 sugerem redundância dos itens da escala e necessidade de redução do instrumento^26^.

Este estudo fornece evidências para caracterizar o SRQ-20 como um instrumento multidimensional, com dimensões que variaram segundo os diferentes grupos ocupacionais investigados. As estimativas de α não podem ser interpretadas como propriedade do instrumento, uma vez que estão condicionadas aos escores da escala em uma determinada população^24^.

Em geral, medidas unidimensionais apresentam elevados níveis de consistência interna (alta homogeneidade). Entretanto, altos valores de consistência interna representam condições necessárias, mas não suficientes, para garantir unicidade de uma escala^5^. Assim, os instrumentos multidimensionais que apresentam elevados níveis de consistência interna de uma determinada medida mostram que, apesar de existirem diferentes dimensões, os itens componentes estão fortemente inter-relacionados^16^.

Os valores de α podem subestimar a verdadeira consistência interna das medidas de um instrumento multidimensional, devido à estimativa de α sugerir discriminação equivalente entre os itens do questionário^18^ ^,^ ^19^ ^,^ ^b^. Escalas congenéricas, caracterizadas pela correlação dos itens entre si, também sofrem efeito da subestimação dos valores de alpha^10^. Para instrumentos multidimensionais, o α estratificado tem apresentado melhor desempenho das estimativas que os estimadores convencionais, apesar de as diferenças não serem tão expressivas^b^. Por isso, o princípio de tau equivalência deve ser considerado ao analisar as estimativas de α de Cronbach entre instrumentos multidimensionais. Nesses casos, a pressuposição de que cada item do teste mede o mesmo traço latente em um instrumento é violada pelas dimensões extraídas pela análise fatorial. Dessa forma, os valores de α das medidas de um instrumento multidimensional serão subestimadas^26^.

No contexto das avaliações psicométricas, destaca-se o debate sobre a superioridade das medidas utilizadas como critérios de confiabilidade^3^. As medidas de α de Cronbach são amplamente utilizadas de forma acrítica e muitas vezes consideradas referência para confiabilidade de escalas. Entretanto, é necessário aprofundar-se na análise dos valores de α e comparar os índices com base na repetição de medidas (teste-reteste)^10^.

Limitações operacionais e teóricas traduzem baixo limiar de consistência interna para as estimativas produzidas pelo α de Cronbach. Além disso, subsistem as dificuldades de interpretação e julgamento das suas medidas. As estimativas do alpha se correlacionam com outras estatísticas, o que pode confundir resultados quando valores muito baixos e muito altos deste coeficiente são encontrados em instrumentos unidimensionais ou multidimensionais. Assim, são necessárias informações adicionais para avaliar as estimativas de α, isoladamente, como medida de consistência interna^23^.

Apesar do uso extensivo do α de Cronbach na avaliação da consistência interna do SRQ-20, ainda é recente a discussão sobre as reais implicações de suas medidas para os instrumentos multidimensionais. São necessários estudos que avaliem criticamente as estimativas de α de Cronbach, comparando-as com métodos alternativos e informações adicionais que permitam melhor dimensionamento da precisão dos instrumentos de mensuração de construtos latentes, como no caso dos transtornos mentais.

## Footnotes

a World Health Organization, Division of Mental Health. User’s guide to self-reporting questionnaire (SRQ). Geneva: World Health Organization; 1994.

b Widhiarso W. Estimate reliability measurement for multidimensional scales. Social Science Research Network; 2009 [citado 2015 jul 28]. Disponível em: http://papers.ssrn.com/sol3/papers.cfm?abstract_id=1597532
